# Modeling Evasive Response Bias in Randomized Response: Cheater Detection Versus Self-protective No-Saying

**DOI:** 10.1007/s11336-024-10000-x

**Published:** 2024-08-30

**Authors:** Khadiga H. A. Sayed, Maarten J. L. F. Cruyff, Peter G. M. van der Heijden

**Affiliations:** 1 Department of Methodology and Statistics, Utrecht University, Padualaan 14, 3584 CH, Utrecht, The Netherlands; 2 Department of Statistics, Faculty of Economics and Political Science, Cairo University, Cairo, Egypt; 3 Department of Social Statistics and Demography, University of Southampton, Southampton, UK

**Keywords:** sensitive questions, ever/last year, anabolics, doping

## Abstract

Randomized response is an interview technique for sensitive questions designed to eliminate evasive response bias. Since this elimination is only partially successful, two models have been proposed for modeling evasive response bias: the cheater detection model for a design with two sub-samples with different randomization probabilities and the self-protective no sayers model for a design with multiple sensitive questions. This paper shows the correspondence between these models, and introduces models for the new, hybrid “ever/last year” design that account for self-protective no saying and cheating. The model for one set of ever/last year questions has a degree of freedom that can be used for the inclusion of a response bias parameter. Models with multiple degrees of freedom are introduced for extensions of the design with a third randomized response question and a second set of ever/last year questions. The models are illustrated with two surveys on doping use. We conclude with a discussion of the pros and cons of the ever/last year design and its potential for future research.

Randomized response (RR) is an indirect survey method introduced by Warner (1965) to eliminate evasive response to sensitive questions. RR involves the use of a randomizer (e.g., a die or a spinner) that adds random noise to the responses so that they do not reveal the respondent’s true response, i.e., the truthful response that would have been given to a direct question. Several comparative validation studies [e.g., Umesh and Peterson (1991), Lamb and Stem (1978), Tracy and Fox (1981), Moshagen et al. (2014), Hoffmann et al. (2015), Lara et al. (2006)] and two meta-analyses (Lensvelt-Mulders et al., 2005; Sagoe et al., 2021) have shown that RR tends to yield more valid responses than direct questioning. In general, RR yields higher prevalence estimates when the sensitive attribute is socially undesirable (the “more-is-better” criterion) and lower prevalence estimates when the sensitive attribute is socially desirable (the “less-is-better” criterion) (Mieth et al., 2021; Meisters et al., 2022a).

Although RR protects the respondents’ privacy, several studies showed that RR does not fully eliminate evasive response behavior (Edgell, 1982; Böckenholt et al., 2009; Wolter and Preisendörfer, 2013; Höglinger et al., 2016; John et al., 2018; van der Heijden et al., 2000). For example, in a study by van der Heijden et al. (2000) all respondents were known to have committed fraud, but RR yielded a prevalence estimate around 50%, and in a study by Edgell (1982), where the outcomes of the randomizer were predetermined, 25% of respondents gave an evasive “no” answer while the randomizer required them to answer “yes.” In a qualitative study of the forced response design (Boruch, 1971) by Boeije and Lensvelt-Mulders (2002), some respondents admitted to have edited their responses because they did not want to falsely incriminate themselves by giving a forced “yes” response.

Since evasive responses bias the prevalence estimates, it is important to correct for them. The problem with RR designs like that of Warner, forced response (Boruch, 1971), the unrelated question (Greenberg et al., 1969), and the crosswise design (Tian and Tang, 2013), is that their statistical models are saturated, because they have only one non-redundant randomized response proportion (that of the “yes” responses in the sample) to estimate the prevalence of the sensitive attribute. As a consequence, an additional parameter accounting for evasive response bias would not be identified. To model evasive response bias, degree(s) of freedom need to be generated for the inclusion of the additional parameter. In this paper, we compare three different designs that generate the necessary degree of freedom, and two different models that use this degree to model evasive response bias.

The sub-samples design in combination with the cheater detection model (CDM) was introduced by Clark and Desharnais (1998). This design generates a degree of freedom by splitting the sample in two non-overlapping sub-samples with different randomization probabilities, and the CDM estimates the prevalence of (i) instruction-adherent carriers of the sensitive attribute, (ii) instruction-adherent non-carriers of the sensitive attribute, and (iii) cheaters, i.e., respondents with unknown true response who give the evasive answer irrespective of the outcome of the randomizer. The prevalence estimate of the sensitive attribute therefore has a lower and upper bound, respectively, given by the estimate of the instruction-adherent carrier and the sum of the estimates of the instruction-adherent carriers and the cheaters. For details of the statistical properties of this model, see Feth et al. (2017). The CDM was used in combination with the forced response design by Clark and Desharnais (1998), but has also been used in combination with the unrelated question and triangular designs (Ostapczuk et al., 2009; Reiber et al., 2020, 2022; Meisters et al., 2022b). Topics that have been investigated with the CDM include doping use by elite and recreational athletes (Christiansen et al., 2023; Elbe and Pitsch, 2018; Pitsch and Emrich, 2011; Petróczi et al., 2022; Schröter et al., 2016; Fincoeur and Pitsch, 2017; Frenger et al., 2016), cheating in examinations (Ostapczuk et al., 2009), medication non-adherence (Ostapczuk et al., 2011), intimate partner violence during the COVID-19 pandemic (Reiber et al., 2022), and social welfare fraud (van den Hout et al., 2010a). The latter used a dual sampling scheme with RR questions in one sub-sample and direct questions in the other. The developed extended crosswise model (Heck et al., 2018) that has recently received much attention also uses the sub-samples design, but because it does not use the response categories “yes/no” it does do not lend itself for the estimation of cheating/SP-no saying [for details, see Heck et al. (2018), Sayed et al. (2022)], and its discussion is therefore beyond the scope of this paper.

The SP-no model was introduced by Böckenholt and van der Heijden (2007) for the multiple questions design. This design consists of $p\ge 2$ dichotomous sensitive questions inquiring about different sensitive attributes. The SP-no model analyses the $2^p$ randomized response profiles under the assumption that the probabilities of the $2^p$ true response profiles can be described by a constrained multivariate distribution. This constraint generates the degree of freedom necessary to account for the presence of self-protective no sayers (SP-no sayers) who give an evasive “no” response to all questions, irrespective the outcome of the randomizer. In contrast to the CDM, the SP-no model does not treat the SP-no sayers as a separate category alongside the carriers and non-carriers, but it corrects the prevalence estimates of carriers and non-carriers for SP-no saying. The SP-no model has been used with various constrained multivariate distributions, including an item response theory (IRT) variant (Böckenholt and van der Heijden, 2007; Böckenholt et al., 2009; Fox and Meijer, 2008; De Jong et al., 2010), a log-linear variant (Cruyff et al., 2007; van den Hout et al., 2010b) and a zero-inflated Poisson variant (Cruyff et al., 2008a, b), and it has also been used with a mixture of dichotomous and polytomous questions (Fox et al., 2013; Cruyff et al., 2016). The log-linear and IRT models can generate more than one degree of freedom, which makes it possible to estimate item-specific SP-no parameters that correspond to the sensitivity of the items [see, Böckenholt et al. (2009)]. The SP-no model has been used for prevalence estimation of such topics as social welfare fraud (Böckenholt and van der Heijden, 2007; Böckenholt et al., 2009; Cruyff et al., 2007, 2008a, b, 2016), smoking behavior (Fox et al., 2013), attitudes toward academic learning (Fox and Meijer, 2008), and sexual attitudes (De Jong et al., 2010).

The CDM and SP-no models appear to be different approaches to modeling evasive response bias, with the former corresponding to the sub-samples design and the latter to the multiple questions design. Although van den Hout et al. (2010a) has briefly commented on the correspondence between the models, little is known about the similarities and differences between the two models. This makes it difficult for researchers who want to conduct an RR survey and correct for response bias to select the appropriate model. This paper sheds more light on this issue by showing the correspondence between the parameters of both models and derives such models for the ever/last year design.

This paper also introduces a new design that can serve as an alternative to the existing designs for detecting evasive response bias. Recently, Sayed et al. (2023) proposed a model for the RR design with “ever” and “last year” questions, the former asking about the presence of a sensitive attribute during the respondent’s lifetime and the latter about its presence during the last year. This design was originally developed to investigate the prevalence of a sensitive attribute over time, but it has some favorable properties that are useful even if the primary interest is not in the prevalence estimates over time. The model for this design estimates the prevalence of non-carriers, former carriers and last year carriers of the sensitive attribute from the four observed randomized response profiles, and therefore has one degree of freedom. An important advantage of this model is that it estimates the prevalence of the last year carriers more efficiently than when only the “last year” question is asked. Another advantage is that there is no need for splitting the sample in two sub-samples with different randomization probabilities nor for assuming a constrained multivariate distribution for the true response profiles to generate the degree of freedom for the estimation of response bias, because this degree of freedom is already available. The ever/last year design has been applied in two studies on doping use in The Netherlands (De Hon et al., 2015; Hilkens et al., 2021), but it did not include a parameter to account for evasive response bias.

This paper introduces response bias models for the ever/last year design with a single set of ever/last year questions, and extension to an additional third dichotomous randomized response question and a second set of ever/last year questions. The benefits of these extensions are twofold. Firstly, they increase the power to detect response biases. Secondly, they increase the degrees of freedom, which allows for the inclusion of multiple parameters to test different assumptions about response bias. These models are applied to data from two Dutch surveys with ever/last year question on the use of anabolic steroids and SARMs, and two sets of ever/last year questions on the use of anabolics and blood manipulations.

The paper is structured as follows. Section 1 reviews the CDM for the sub-samples design and SP-no model for the multiple question design and shows the correspondence between these models by applying the SP-no model to the sub-samples design and the CDM to the multiple questions design. In Sect. 2, we derive the CDM and the SP-no model for the ever/last year design. Section 3 presents the maximum likelihood estimators of the model parameters. Section 4 investigates the power to detect cheating/SP-no saying in the design with one set of ever/last year and designs with an additional third question and a second set of ever/last year questions. Section 5 presents the results of analyses of the data of a survey with ever/last year questions about anabolic steroids and a dichotomous RR question about SARMs use by gym users in the Netherlands and of a survey with ever/last year questions about both the use of androgenic anabolics and blood manipulations by Dutch elite athletes. The analyses include prevalence estimation of these two types of doping and of cheating/SP-no saying. Section 6 discusses the pros and cons of both approaches for modeling evasive response bias and some limitations. Section 7 ends the paper with discussion of the pros and cons of the various designs and suggestions for future research.

## Correspondence between the CDM and SP-no model

In this section, we review the CDM for the sub-samples design and the SP-no model for the multiple questions design, and then show that the SP-no model can also be applied to the sub-samples design, and that the CDM can also be applied to the multiple questions design. We start the section with an introduction of the matrix notation for dichotomous, saturated randomized response models. We use matrix notation because it provides a visualization of the models that facilitates their interpretation. In Sect. 3, we show that the use of matrix notation also greatly facilitates parameter estimation.

Consider a design with a single dichotomous sensitive question. Let $\pi _t$ denote the probability of the true response and $\pi ^*_r$ the probability of the randomized response, for $r,t\in \{n=no,y=yes\}$, and let $\varvec{P}_{2\times 2}$ be the $2\times 2$ transition matrix with entries $p_{r|t}$ denoting the conditional randomization probabilities of observing randomized response *r* given true response *t*. In matrix notation, the model $\varvec{\pi }^*=\varvec{P}_{2\times 2}\varvec{\pi }$ for this design is given by

1.1 $$\begin{aligned} \left( \begin{array}{c} \pi ^*_n\\ \pi ^*_y \end{array}\right) = \left( \begin{array}{cc} p_{n|n} & p_{n|y}\\ p_{y|n} & p_{y|y} \end{array}\right) \left( \begin{array}{c} \pi _n\\ \pi _y \end{array}\right) = \left( \begin{array}{c} p_{n|n}\pi _n+p_{n|y}\pi _y \\ p_{y|n}\pi _n+p_{y|y}\pi _y \end{array}\right) , \end{aligned}$$

for $p_{n|n}\ne p_{y|n}$ and $p_{n|y}\ne p_{y|y}$ (Chaudhuri and Mukerjee, 1988; van den Hout and van der Heijden, 2002).

To simplify the notation of the transition matrices, we will from hereon restrict the model derivations to designs with symmetrical randomization probabilities $p=p_{y|y}=p_{n|n}$ and $q=p_{y|n}=p_{n|y}$, for $p+q=1$ and $p\ne {q}$, so that the probability that a carrier answers *y* is equal to the probability that a non-carrier answers *n*.

The transition matrix defines the statistical properties of the model. For example, for Warner’s design with probability.8 of answering the sensitive question $p=.8$ and $q=.2$, and for the unrelated question design (Greenberg et al., 1969) with probability 0.6 of answering the sensitive question and probability 0.5 of answering “yes” to the unrelated question, $p=.6+.5 \times .4=.8$ and $q=.5\times .4$, so that the models for both designs have the same transition matrix

1.2 $$\begin{aligned} \varvec{P}_{2\times 2} = \left( \begin{array}{cc} p & q\\ q & p \end{array}\right) = \left( \begin{array}{cc}.8 & .2\\ .2 & .8 \end{array}\right) , \end{aligned}$$

which shows that these designs are mathematically equivalent. The efficiency of a design is determined by diagonal entries of the transition matrix; the closer to 1 the higher its efficiency ($p=1$ corresponds to the direct question design). For $p=.5$, the design is uninformative because it results in the randomized response probabilities $\pi ^*_n=\pi ^*_y=.5$, irrespective of the prevalence of the sensitive attribute.

For a design with two sensitive questions *A* and *B* and $r_{AB},t_{AB}\in \{nn, ny,yn,yy\}$, respectively, denoting randomized and true response profiles, the bivariate model is given by

1.3 $$\begin{aligned} \left( \begin{array}{c} \pi ^*_{nn}\\ \pi ^*_{ny}\\ \pi ^*_{yn}\\ \pi ^*_{yy} \end{array} \right) = \left( \begin{array}{cccc} p^2 & pq & qp & q^2\\ pq & p^2 & q^2 & qp\\ qp & q^2 & p^2 & pq\\ q^2 & qp & pq & p^2 \end{array}\right) \left( \begin{array}{c} \pi _{nn}\\ \pi _{ny}\\ \pi _{yn}\\ \pi _{yy} \end{array} \right) . \end{aligned}$$

where $\varvec{P}_{4\times 4}$ is obtained as the Kronecker $\varvec{P}_{2\times 2}\otimes \varvec{P}_{2\times 2}$. The extension to more than two randomized response variables is straightforward. For example, for three variables *A*, *B*, *C* the response profiles are $r_{ABC},t_{ABC}\in \{nnn, nny, \dots , yyn, yyy\}$ and the transition matrix is $\varvec{P}_{2\times 2}\otimes \varvec{P}_{2\times 2}\otimes \varvec{P}_{2\times 2}$. If *C* is a categorical non-randomized response variable with *p* categories, the transition matrix is obtained by $\varvec{P}_{2\times 2}\otimes \varvec{P}_{2\times 2}\otimes {I}_{p\times {p}}$, where $I_{p\times {p}}$ is the $p\times {p}$ identity matrix (van den Hout and van der Heijden, 2002).

### The CDM for the sub-samples design

The sub-samples design of Clark and Desharnais (1998) splits the sample into two sub-samples $s\in \{1,2\}$ with different randomization probabilities $p_s$ and $q_s$. The CDM for this design is formulated in terms of the conditional randomized response probabilities $\pi ^*_{r|s}$ denoting the probability of observing randomized response *r* given membership of sub-sample *s*, for $\sum _r\pi ^*_{r|s}=1$. The advantage of this formulation is that the sub-sample sizes $n_s$ drop out the equation, which simplifies notation and interpretation. The model assuming instruction-adherence is

1.4 $$\begin{aligned} \left( \begin{array}{c} \pi ^*_{n|1}\\ \pi ^*_{y|1}\\ \pi ^*_{n|2}\\ \pi ^*_{y|2} \end{array}\right) = \left( \begin{array}{ccc} p_1 & q_1\\ q_1 & p_1\\ q_2 & p_2\\ p_2 & q_2 \end{array}\right) \left( \begin{array}{c} \pi _n\\ \pi _y \end{array}\right) , \end{aligned}$$

A common choice for $p_s$ is to set $p_1=p_2$, resulting in complementary randomization probabilities $p=p_{n|n}=p_{y|y}$ for sub-sample 1 and $p=p_{n|y}=p_{y|n}$ for sub-sample 2. In the remainder of this paper we will assume that the randomization probabilities are complementary and drop the subscript *s* from $p_s$ and $q_s$.

This model has one degree of freedom, because there are two non-redundant randomized response probabilities to estimate one non-redundant true response probability. To illustrate the model, consider a design with the two statements “I used doping” and “I never used doping,” and that respondents in sub-sample 1 answer the first statement with probability.8 and the second with probability.2, while respondents in sub-sample 2 answer the first statement with probability.2 and the second with probability.8. With a true prevalence of doping use $\pi _y=.2$ and randomization probability $p=1-q=.8$, the model is given by

$$\begin{aligned} \left( \begin{array}{c}.68\\ .32\\ .32\\ .68 \end{array}\right) = \left( \begin{array}{ccc}.8 & .2\\ .2 & .8\\ .2 & .8\\ .8 & .2 \end{array}\right) \left( \begin{array}{c}.8\\ .2 \end{array}\right) . \end{aligned}$$

This example shows that in case of instruction-adherence $\pi ^*_{n|1}+\pi ^*_{n|2}=1$ and $\pi ^*_{y|1}+\pi ^*_{y|2}=1$.

The CDM postulates the presence of “cheaters,” i.e., respondents who answer “no” irrespective of the outcome of the randomizer and for whom the true response is unknown. Consequently, the CDM distinguishes between three true responses $t\in \{n,y,c\}$, with *n* denoting the instruction-adherent non-carriers, *y* the instruction-adherent carriers, and *c* the cheaters with unknown true response. By replacing $\varvec{\pi }=(\pi _n,\pi _y)'$ of model (1.4) by $\varvec{\tau }=(\tau _n, \tau _y, \tau _c)'$, for $\tau _y + \tau _n + \tau _c = 1$, the CDM is given by

1.5 $$\begin{aligned} \left( \begin{array}{c} \pi ^*_{n|1}\\ \pi ^*_{y|1}\\ \pi ^*_{n|2}\\ \pi ^*_{y|2} \end{array}\right) = \left( \begin{array}{ccc} p & q & 1\\ q & p & 0\\ q & p & 1\\ p & q & 0 \end{array}\right) \left( \begin{array}{c} \tau _n\\ \tau _y\\ \tau _c \end{array}\right) , \end{aligned}$$

where the third column of the transition matrix corresponds to the conditional randomization probabilities for the cheaters, for whom $p_{n|c}=1$ and $p_{y|c}=0$ in both sub-samples.

As an example, consider the randomized response probabilities $\varvec{\pi }^*=(.7,.3,.4,.6)'$, which indicate the presence of cheaters because $\pi ^*_{n|1}+\pi ^*_{n|2}=.7+.4>1$. The corresponding true response probabilities are $\varvec{\tau }=(.7,.2,.1)$, so that the prevalence of doping has a lower bound of $\tau _y=.2$ (the instruction-adherent carriers) and an upper bound $\tau _y+\tau _c=.2 +.1 =.3$ (the instruction-adherent carriers and the cheaters).

### The SP-no model for the multiple questions design

To formulate a general SP-no model for a design with two sensitive questions inquiring about two different sensitive attributes, we extend model (1.3) with the parameters $\theta _{t_{AB}}$, denoting the probability of SP-no saying by respondents with true response profile $t_{AB}$. The model is

1.6 $$\begin{aligned} \left( \begin{array}{c} \pi ^*_{nn}\\ \pi ^*_{ny}\\ \pi ^*_{yn}\\ \pi ^*_{yy} \end{array}\right) = \left( \begin{array}{cccc} p^2 & pq & qp & q^2\\ pq & p^2 & q^2 & qp\\ qp & q^2 & p^2 & pq\\ q^2 & qp & pq & p^2 \end{array}\right) \left( \begin{array}{c} (1-\theta _{nn})\pi _{nn}\\ (1-\theta _{ny})\pi _{ny}\\ (1-\theta _{yn})\pi _{yn}\\ (1-\theta _{yy})\pi _{yy} \end{array}\right) + \left( \begin{array}{c} \theta _{nn}\pi _{nn}+\theta _{ny}\pi _{ny}+\theta _{yn}\pi _{yn}+\theta _{yy}\pi _{yy}\\ 0\\ 0\\ 0 \end{array}\right) , \nonumber \\ \end{aligned}$$

where $(1-\theta _{t_{AB}})\pi _{t_{AB}}$ denotes the probability that respondents with true response profile $t_{AB}$ are instruction-adherent and the vector at the right-hand side of the model denotes the total probability of observing an SP-no response.

With four randomized response probabilities $\pi ^*_{r_{AB}}$ and the eight parameters $\theta _{t_{AB}}$ and $\pi _{t_{AB}}$ to be estimated, the model is obviously over-parameterized. The model can be identified by i) assuming an equal SP-no probability for all true response profiles and ii) assuming independence of the two sensitive attributes by formulating the log-linear independence model (*A*, *B*) given by $\log \pi _{t_{AB}}=\lambda +\lambda ^A_{t_A}+\lambda ^B_{t_B}$ for the probabilities of the true response profiles. Using dummy coding for the log-linear model with the true responses $t_A,t_B=n$ as reference category, the model becomes

1.7 $$\begin{aligned} \left( \begin{array}{c} \pi ^*_{nn}\\ \pi ^*_{ny}\\ \pi ^*_{yn}\\ \pi ^*_{yy} \end{array}\right) = (1-\theta ) \left( \begin{array}{cccc} p^2 & pq & qp & q^2\\ pq & p^2 & q^2 & qp\\ qp & q^2 & p^2 & pq\\ q^2 & qp & pq & p^2 \end{array}\right) \left( \begin{array}{c} e^{\lambda }\\ e^{\lambda +\lambda ^B_{y}}\\ e^{\lambda +\lambda ^A_{y}}\\ e^{\lambda +\lambda ^A_{y}+\lambda ^B_{y}} \end{array}\right) + \theta \left( \begin{array}{c} 1\\ 0\\ 0\\ 0 \end{array}\right) . \end{aligned}$$

This parameterization shows that the SP-no model can be interpreted as a mixture model, with $1-\theta$ the probability to the latent class of instruction-adherent respondents, and $\theta$ the probability of the latent class of SP-no sayers. The vector $(1,0,0,0)'$ can be interpreted as the randomization probabilities of the SP-no sayers, which are 1 for the randomized response profile *nn* and 0 otherwise.

The validity of this model depends on the strong assumption that the two sensitive attributes are independent. If the sensitive attributes are not independent, the parameter estimates of model (1.7) will be biased. To illustrate, consider the true response probability vector $\varvec{\pi }=(.4,.1,.4,.1)'$ implying independence of the two sensitive attributes. With a prevalence $\theta =.2$ of SP-no sayers and $p=.8$, the model yields the unbiased estimates $\hat{\varvec{\pi }}=(.4,.1,.4,.1)'$ and $\hat{\theta }=.2$. However, for the vector $\varvec{\pi }=(.4,.3,.2,.1)'$ implying dependence of the two sensitive attributes, the estimates $\hat{\varvec{\pi }}=(.46,.27,.17,.10)'$ and $\hat{\theta }=.164$ are biased.

The independence assumption can be relaxed by asking three questions *A*, *B*, *C* and formulating the log-linear model (*AB*, *AC*, *BC*) given by $\log \pi _{t_{ABC}}=\lambda +\lambda ^A_{t_A}+\lambda ^B_{t_B}+\lambda ^C_{t_C}+\lambda ^{AB}_{t_{AB}}+\lambda ^{AC}_{t_{AC}}+\lambda ^{BC}_{t_{BC}}$. This model constrains the three-factor interaction $\lambda ^{ABC}_{t_{ABC}}$ to zero to obtain the degree of freedom for model identification, but includes all pairwise interactions. With three or more questions, it also becomes possible to specify an IRT model as introduced by Böckenholt and van der Heijden (2007).

On first sight the CDM and SP-no models may appear to be incompatible, but in the next two sections we show that these models are two sides of the same coin by writing the parameters of one in terms of the parameters of the other.

### The SP-no model for the sub-samples design

To apply the SP-no model to the sub-samples design, we use the general formulation (1.6) with separate probabilities $\theta _y$ for the carriers an $\theta _n$ for the non-carriers. This yields the model

1.8 $$\begin{aligned} \left( \begin{array}{c} \pi ^*_{n|1}\\ \pi ^*_{y|1}\\ \pi ^*_{n|2}\\ \pi ^*_{y|2} \end{array}\right) = \left( \begin{array}{cc} p & q\\ q & p\\ q & p\\ p & q \end{array}\right) \left( \begin{array}{c} (1-\theta _n)\pi _{n}\\ (1-\theta _y)\pi _{y} \end{array}\right) + \left( \begin{array}{c} \theta _n\pi _n+\theta _y\pi _y\\ 0\\ \theta _n\pi _n+\theta _y\pi _y\\ 0 \end{array}\right) . \end{aligned}$$

We have now formulated a model for the sub-samples design for which $\pi _n+\pi _y=1$, and the prevalence of the cheaters $\tau _c$ is replaced by the SP-no sayer parameters $\theta _y$ and $\theta _n$. This allows us to make different assumptions with respect to the true responses of the SP-no sayers and enables us to investigate the correspondence between the parameters of the CDM (1.5) and the SP-no model (1.8). Table 1 summarizes this correspondence for $\theta _n=\theta _y$, $\theta _n=0$ and $\theta _y=0$. For the derivations of these equality relations we refer the Appendix A on OSF (https://osf.io/autr5/?view_only=2af4b338b9be45cd8657f926438c5f93).

**Table 1:** Correspondence between the parameters of the CDM and SP-no model.

	Parameters $\theta$ of the SP-no model for the sub-samples design		
	$\theta _y=\theta _n$	$\theta _n=0$	$\theta _y=0$
$\pi _y$	$\tau _y/(1-\tau _c)$	$\tau _y+\tau _c$	$\tau _y$
$\pi _n$	$\tau _n/(1-\tau _c)$	$\tau _n$	$\tau _n+\tau _c$
$\theta$	$\tau _c$	$\tau _c/(1-\tau _n)$	$\tau _c/(1-\tau _y)$

The table shows that for $\theta _n=\theta _y$ the prevalence of cheaters is equal to the prevalence of SP-no sayers, and that the prevalence of carriers $\pi _y$ is equal to the conditional probability of the adherent carriers $\tau _y$ given the non-cheaters $1-\tau _c$. For $\theta _n=0$ and $\theta _y=0$, the prevalence of carriers $\pi _y$, respectively, corresponds to the upper and lower bound of the prevalence of the carriers under the CDM, and $\theta$ corresponds to the respective conditional probabilities of cheating given cheaters and adherent carriers and of cheating given cheaters and adherent non-carriers.

### The CDM for the multiple questions design

To apply the CDM to the multiple questions design, we replace the vector $\varvec{\pi }=(\pi _{nn}, \pi _{ny},\pi _{yn},\pi _{yy})'$ of the SP-no model (1.7) by the vector $\varvec{\tau }=(\tau _{nn}, \tau _{ny},\tau _{yn},\tau _{yy}, \tau _c)'$ and formulate the log-linear independence model $\log \tau _{t_{AB}}=\lambda +\lambda ^A_{t_A}+\lambda ^B_{t_B}$ for the true response profiles in $\varvec{\tau }$ corresponding to the instruction-adherent respondents. The vector denoting the latent class of SP-no sayers in (1.7) is replaced by a fifth column in the transition matrix with the randomization probabilities of the cheaters. The model is then given by

1.9 $$\begin{aligned} \left( \begin{array}{c} \pi ^*_{nn}\\ \pi ^*_{ny}\\ \pi ^*_{yn}\\ \pi ^*_{yy} \end{array}\right) = \left( \begin{array}{ccccc} p^2 & pq & qp & q^2 & 1\\ pq & p^2 & q^2 & qp & 0\\ qp & q^2 & p^2 & pq & 0\\ q^2 & qp & pq & p^2 & 0 \end{array}\right) \left( \begin{array}{l} (1-\tau _c)e^{\lambda }\\ (1-\tau _c)e^{\lambda + \lambda ^B_{y}}\\ (1-\tau _c)e^{\lambda + \lambda ^A_{y}}\\ (1-\tau _c)e^{\lambda + \lambda ^A_{y} + \lambda ^B_{y}}\\ \tau _c \end{array}\right) . \end{aligned}$$

The correspondence between the parameters of SP-no model (1.7) and the CDM (1.9) can no longer be established by assuming that the SP-no sayers of model (1.7) are either carriers or non-carriers, because there is now a mixture of carriers and non-carriers on the two sensitive attributes. Under the assumption that all four true response profiles have the same probability of SP-no saying, the correspondence is analogous to that described in the first column of Table 1, i.e., $\theta =\tau _c$ and $\pi _{t_{AB}}=\tau _{t_{AB}}/(1-\tau _c)$.

## Models for ever/last year designs

The ever/last year design is characterized by two questions about the same sensitive attribute; one about its presence during the respondent’s lifetime, and one about its presence in the last year. It can be considered a hybrid of the cheater detection and the multiple questions designs. Like the sub-samples design a single sensitive attribute is queried, but like the multiple questions design it employs multiple (in this case two) sensitive questions. The design makes it possible to identify last year, former and non-carriers of the sensitive attribute. The advantages of this design over a design with a single question design with the three responses “never,” “former” and “last year” are that the model for the ever/last year design has a degree of freedom and that the prevalence of the last year carriers is estimated with higher precision (Sayed et al. (2023)).

In this section, we consider models for one set of ever/last year questions, for one set of ever/last year question and a third, dichotomous randomized response question, and for two sets of ever/last year questions. We introduce the SP-no and CDM versions of these models with the parameters $\theta$ and $\tau _c$, and for the SP-no models with more than one degree of freedom, we include an additional parameter that accounts for evasive responses to the last year question by last year carriers.

### A single set of ever/last year questions

The model for one set of ever/last year questions comes with one degree of freedom. This degree of freedom is due to the fact that the true response profile *ny* of never been carrier while having been carrier during the last year is impossible. As a consequence, the parameter $\pi _{ny}$ and the second column of the $\varvec{P}_{4x4}$ transition matrix of model (1.3) are redundant. The null model for this design is given by

2.1 $$\begin{aligned} \left( \begin{array}{c} \pi ^*_{nn}\\ \pi ^*_{ny}\\ \pi ^*_{yn}\\ \pi ^*_{yy} \end{array}\right) = \left( \begin{array}{ccc} p^2 & qp & q^2\\ pq & q^2 & qp\\ qp & p^2 & pq\\ q^2 & pq & p^2 \end{array}\right) \left( \begin{array}{c} \pi _{nn}\\ \pi _{yn}\\ \pi _{yy} \end{array}\right) . \end{aligned}$$

The true response profiles $t\in \{nn, yn, yy\}$ are, respectively, interpreted as the non-carriers (those who never carried the sensitive attribute), the former carriers (those who have once carried the sensitive attribute, but not in the last year), and the last year carriers (those who carried the sensitive attribute in the last year and possibly before).

The CDM version of this model is

2.2 $$\begin{aligned} \left( \begin{array}{c} \pi ^*_{nn}\\ \pi ^*_{ny}\\ \pi ^*_{yn}\\ \pi ^*_{yy} \end{array}\right) = \left( \begin{array}{cccc} p^2 & qp & q^2 & 1\\ pq & q^2 & qp & 0\\ qp & p^2 & pq & 0\\ q^2 & pq & p^2 & 0 \end{array}\right) \left( \begin{array}{c} \tau _{nn}\\ \tau _{yn}\\ \tau _{yy}\\ \tau _c \end{array}\right) , \end{aligned}$$

and the SP-no model is

2.3 $$\begin{aligned} \left( \begin{array}{c} \pi ^*_{nn}\\ \pi ^*_{ny}\\ \pi ^*_{yn}\\ \pi ^*_{yy} \end{array}\right) = (1-\theta ) \left( \begin{array}{lll} p^2 & qp & q^2\\ pq & q^2 & qp\\ qp & p^2 & pq\\ q^2 & pq & p^2 \end{array}\right) \left( \begin{array}{c} \pi _{nn}\\ \pi _{yn}\\ \pi _{yy} \end{array}\right) + \theta \left( \begin{array}{c} 1\\ 0\\ 0\\ 0 \end{array}\right) . \end{aligned}$$

It has been suggested that the ever/last year design may reduce the respondents’ trust in the privacy protection, because two questions on the same sensitive attribute have to be answered. This may especially be the case for last year carriers when they have to answer “yes” to the last year question when they already answered the ever question with “yes.” To account for this kind of response bias, we formulate the SP(last year) model. This model includes, aside from the general SP-no parameter $\theta$, the parameter $\theta _{yy~\rightarrow ~yn}$ denoting the probability that a last year carrier answers *yn* to the ever and last year questions when *yy* was required. This model can be formulated by bringing the $\theta$ and $\theta _{yy~\rightarrow ~yn}$ inside the transition matrix of model 2.2. Given that the *yn* and *yy* response profiles are represented by the third and fourth row of the transition matrix and the last year users by its third column, the transition matrix is given by

2.4 $$\begin{aligned} \varvec{P}_{4\times 3} = \left( \begin{array}{lll} (1-\theta )p^2 +\theta & (1-\theta )qp +\theta & (1-\theta )q^2+\theta \\ (1-\theta )pq & (1-\theta )q^2 & (1-\theta )qp\\ (1-\theta )qp & (1-\theta )p^2 & (1-\theta )pq+\theta _{yy~\rightarrow ~yn}p^2\\ (1-\theta )q^2 & (1-\theta )pq & (1-\theta -\theta _{yy~\rightarrow ~yn})p^2 \end{array}\right) . \end{aligned}$$

For a single set of ever/last year questions this model is not identified, but it can be identified by the inclusion of more questions.

### Model extensions

Model (2.1) is easily extended to more questions. The model for one set of ever/last year questions and a third, dichotomous question is obtained by extending the true and randomized response profiles with the answers to the third question, and constructing the transition matrix $\varvec{P}_{8\times 6}=\varvec{P}_{4\times 3}\otimes \varvec{P}_{2\times 2}$, where $\varvec{P}_{4\times 3}$ is the transition matrix of model (2.1) and $\varvec{P}_{2\times 2}$ is that of the third question. This model has two degrees of freedom. The transition matrix of the model for two sets of ever/last year questions is $\varvec{P}_{16\times 9}=\varvec{P}_{4\times 3}\otimes \varvec{P}_{4\times 3}$, so that this model has 7 degrees of freedom.

The derivation of the SP-no and CDM versions of these models is straightforward. For the SP(last year) model, we replace the parameter $\theta _{yy ~\rightarrow ~ yn}$ in the $4\times 3$ transition matrix (2.4) by $\theta _{yy\cdot ~\rightarrow ~ yn\cdot }$ in the $8\times 6$ transition matrix for the design with a third question, with the dot representing the response to the third question. Analogously, we define the parameters $\theta _{yy\cdot \cdot ~\rightarrow ~yn\cdot \cdot }=\theta _{\cdot \cdot yy~\rightarrow ~\cdot \cdot yn}$ for the design with two sets of ever/last year questions. The R code for constructing these transition matrices is given in Appendix C (https://osf.io/autr5/?view_only=2af4b338b9be45cd8657f926438c5f93).

## Estimation

For the examples presented in this paper, the maximum likelihood estimates (MLEs) of the model parameters are obtained by maximization of the kernel of the log-likelihood

3.1 $$\begin{aligned} \ln \ell (\varvec{\Phi }\mid \varvec{n})=\varvec{n}'\ln \varvec{\pi }^*, \end{aligned}$$

where $\varvec{\Phi }$ is the vector with the model parameters $\varvec{\pi },\varvec{\tau }$ and/or $\varvec{\theta }$, and $\varvec{n}$ the vector with the frequencies of the observed randomized response profiles. Maximization of the log-likelihood may result in negative prevalence estimates of the sensitive attribute(s) (van den Hout and van der Heijden, 2004). To ensure that these parameter estimates are inside the parameter space (0, 1), the parameters $\pi _j$ and $\tau _j$ are estimated via the softmax function $\exp (\beta _j)/\sum _j\exp (\beta _j)$. The sampling variances of $\hat{\pi }_j$ and $\hat{\tau }_j$ are obtained with the delta method (Hoef, 2012). Examples of the use of the delta method can be found in Appendix C (e.g., Section 2.5). The $\theta$ parameter is estimated directly and is therefore allowed to take a negative value.

The Akaike information criterion (AIC), computed as twice the number of model parameters minus twice the log-likelihood, is used as model selection criterion. When comparing models with and without response bias parameters, the model with the lowest AIC is considered to be the best model.

The goodness of fit of the models estimated by maximization of the log-likelihood (3.1) can be evaluated with the asymptotically chi-squared distributed $G^2$ statistic

3.2 $$\begin{aligned} G^2_{(df)}=2\cdot \varvec{n}'\ln (\varvec{n}/\hat{\varvec{n}}) \end{aligned}$$

where $\hat{\varvec{n}}$ is the vector with the fitted randomized response frequencies and *df* the degrees of freedom of the model.

## Power study

This section presents a power study to detect $\theta /\tau _c\in \{0,.05,.1,.15,.2\}$ for the ever/last year designs with one set of ever/last year questions, one set of ever/last year question and a third (dichotomous) question, and two sets of ever/last year questions.

**Fig. 1:**
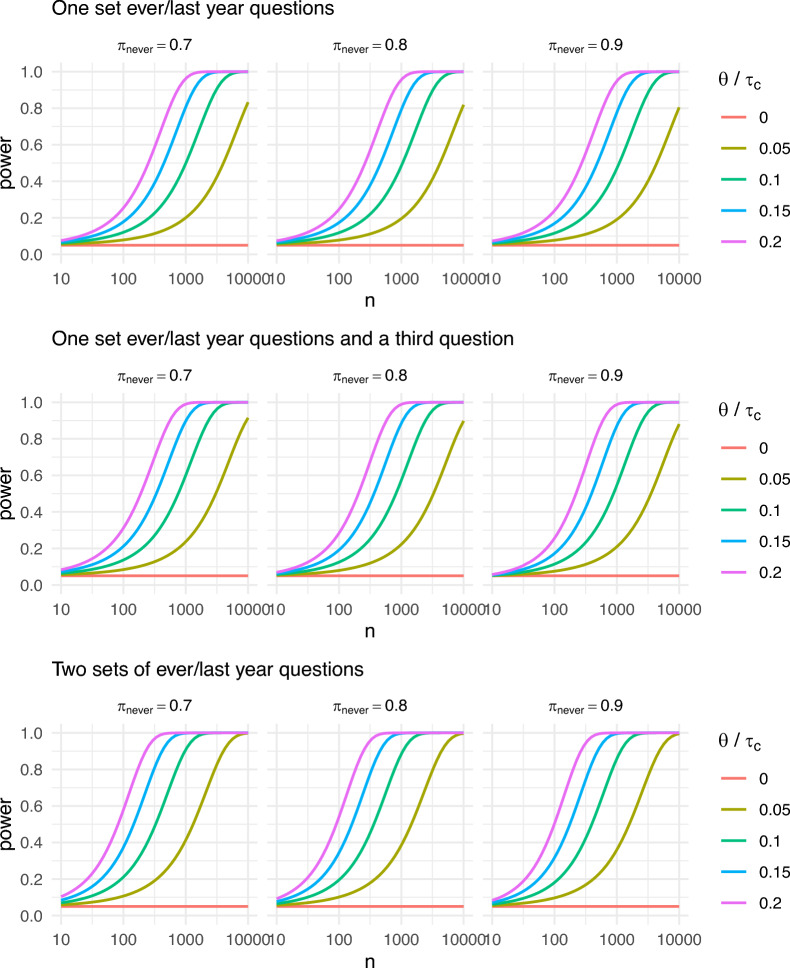
Power curves for detecting $\theta /\tau _c$.

For these three designs, $\pi _{never}$ is defined as the prevalence of respondents with the true response profiles *nn*, *nnn* or *nnnn*, respectively, with the prevalence of remaining true response profiles set to $(1-\pi _{never})/k$, where *k* denotes the number of the remaining true response profiles. The probabilities that the randomized response coincides with the true response is set to $p=5/6$ for each question in the design. The sample sizes *n* are displayed on a logarithmic scale.

The plots show that the power to detect cheating/SP-no saying increases with the number of questions and, to a lesser extent, with smaller values for $\pi _{never}$. For example, to attain a power of 80% to detect of a prevalence of 5% given $\pi _{never}=.9$ requires a sample size around 10, 000 for the design with one set of ever/last year questions, while for the design with two sets of ever/last year questions the required sample size is around 4, 000. For $\pi _{never}\in \{0.7, 0.8\}$, the required sample sizes are slightly smaller. The power increases rapidly as the value for $\theta /\tau _c$ increases. For $\theta /\tau _c=0.1$ and $\pi _{never}=.9$, the required sample sizes are around 2, 500 for one set of ever/last year questions, and around 800 for two sets. Under the most favorable condition that $\pi _{never}=0.7$ and $\theta /\tau _c=0.2$, a sample size around 200 suffices.

We also investigated the power of detecting the $\pi _{former}$ and $\pi _{last~year}$ in the design with one set of ever/last year questions, and for decreasing the probability *p* that the randomized response coincides with the true response. The results show that $\pi _{last~year}$ is estimated more efficiently than when estimated on the basis of a single question, and that the power to detect $\theta /\tau _c$ increases when using $p=2/3$ instead of $p=5/6$. For the power curves of these studies, we refer to Appendix B (https://osf.io/autr5/?view_only=2af4b338b9be45cd8657f926438c5f93).

## Examples

In this section, we present analyses of two online surveys which, aside from demographic and sport-related questions, included randomized response questions on the use of doping. Study I was conducted by the HAN University of Applied Sciences and Utrecht University (Hilkens et al., 2021) among 2, 269 male gym users. In this study the researchers were interested in the prevalence of current and former use of anabolics, and in the lifetime use of SARMs (because SARMs use is a relatively new phenomenon, the researchers found the distinction between former and current use less relevant). The respondents were asked the ever/last year questions “Have you ever/in the last 12 months used anabolic steroids (e.g., Testosterone, Deca, Winstrol, Dianabol, Anavar)?” and the question “Have you ever used SARMs (Selective Androgen Receptor Modulators).” To answer these questions, respondents were shown a circle and a square symbol with the answers “yes” and “no” rapidly changing position. When clicking a *Stop* button the changing of positions stopped, and the respondents were asked to answer “Circle” or “Square” depending on the symbol that contained their true answer. The probability that “yes” ended up in the circle was fixed at 5/6, so that $p_{y|y} = p_{n|n} = 5/6$.

Study II (De Hon et al., 2015) was conducted in 2014 by the Doping Authority Netherlands, which was especially interested in distinguishing between current and former use of doping substances. The sample included 1, 050 Dutch elite athletes, who were asked ever/last year questions about both the use of anabolics and blood manipulations (e.g., EPO). The study employed two different RR techniques, with 535 athletes answering according to the forced response design (Boruch, 1971) and 515 following a procedure similar to that of Study I but with answer categories “A”/“B” instead of “Circle”/“Square.” As in Study I, both RR techniques used $p_{y|y}=p_{n|n}=5/6$. The data of both studies are shown in Table 2. The R code of the analyses is given in Appendix C.

**Table 2:** Observed response frequencies of Studies I and II.

Study I	ever anabolics, last year anabolics, SARMs							
	*nnn*	*nny*	*nyn*	*nyy*	*ynn*	*yny*	*yyn*	*yyy*
	1,220	248	226	61	290	78	114	32
Study II	ever/last year anabolics, ever/last year blood manipulations							
	*nnnn*	*nnny*	*nnyn*	*nnyy*	*nynn*	*nyny*	*nyyn*	*nyyy*
	521	89	93	21	86	23	22	10
	*ynnn*	*ynny*	*ynyn*	*ynyy*	*yynn*	*yyny*	*yyyn*	*yyyy*
	93	18	19	10	22	7	8	8

### Ever/last year use of anabolics

Table 3 shows the prevalence estimates for the ever/last year questions about anabolic steroids use of Study I. The response frequencies can be obtained from Table 2 by collapsing over the third index. The estimates are obtained with models presented in section 2.1. All three models fit the data adequately, but the AIC prefers the null model over the response bias models, so that these models do not provide significant evidence for SP-no/cheating. The null model estimates a prevalence of 4.3% of former user of anabolics, and 4.7% of last year users.

**Table 3:** Prevalence estimates of anabolics (A) of Study I.

Parameters	Null model	SP-no	CDM
$\pi \tau _{nn}$ (never A)	.911 (.018)	.894 (.021)	.852 (.054)
$\pi \tau _{yn}$ (former A)	.043 (.014)	.056 (.019)	.054 (.017)
$\pi \tau _{yy}$ (last year A)	.047 (.008)	.050 (.009)	.048 (.008)
$\theta /\tau _{c}$		.046 (.041)	.046 (.041)
goodness of fit	$G^2_{(1)}=1.22, ~p=.27$	$G^2_{(0)}=0$	$G^2_{(0)}=0$
AIC	4614.4	4615.1	4615.1

### Ever/last year use of anabolics and ever use of SARMs

Table 4 presents the prevalence estimates of the models presented in Sect. 2.2 for the ever/last year questions about anabolics and the ever question about SARMs of Study I. For these data, the SP(last year) model has been included in the analysis. The null, SP-no and CDM models exhibit an adequate fit. The AIC prefers the SP-no and CDM models, which yield a prevalence estimate of 6.4% for cheating/SP-no saying. And although the SP(last year) model yields significant estimates for both $\theta$ parameters, the model is not preferred on the basis of the AIC.

**Table 4:** Parameter estimates of anabolics (A) and SARMs (S) of Study I.

Parameters	Null model	SP-no	SP(last year)	CDM
$\pi /\tau _{nnn}$ (never A, never S)	.901 (.018)	.855 (.034)	.827 (.041)	.800 (.061)
$\pi /\tau _{nny}$ (never A, ever S)	.009 (.013)	.031 (.020)	.045 (.022)	.029 (.017)
$\pi /\tau _{ynn}$ (former A, never S)	.030 (.016)	.053 (.021)	.001 (.014)	.049 (.019)
$\pi /\tau _{yny}$ (former A, ever S)	.012 (.008)	.010 (.009)	.000 (.004)	.009 (.009)
$\pi /\tau _{yyn}$ (last year A, never S)	.041 (.009)	.046 (.010)	.114 (.031)	.043 (.009)
$\pi /\tau _{yyy}$ (last year A, ever S)	.005 (.005)	.005 (.006)	.013 (.009)	.005 (.005)
$\theta /\tau _{c}\quad ~$		.064 (.037)	.099 (.043)	.064 (.037)
$\theta _{yy\cdot ~\rightarrow ~yn\cdot }$			.419 (.067)	
goodness of fit	$G^2_{(2)}=3.86$	$G^2_{(1)}=0.97$	$G^2_{(0)}=0.17$	$G^2_{(1)}=0.97$
	$p=.14$	$p=.32$		$p=.32$
AIC	6785.2	6784.3	6785.5	6784.3

### Ever/last year use of anabolics (A) and blood manipulations (B).

Table 5 presents the parameter estimates of the models for the two sets of ever/last year questions of Study II. All four models exhibit an adequate fit, but the AIC prefers the SP-no/CDM. These models yield a prevalence estimate of 7.1% for SP-no/cheating. The inclusion of the parameters $\theta _{yy\cdot \cdot ~\rightarrow ~yn\cdot \cdot }=\theta _{\cdot \cdot yy~\rightarrow ~\cdot \cdot yn}$ of the SP(last year) model does not improve the goodness of fit of these models.

**Table 5:** Parameter estimates of anabolics and blood manipulations of Study II.

Parameters	Null model	SP-no	SP(last year)	CDM
$\pi /\tau _{nnnn}$ (never A, never B)	.966 (.012)	.946 (.035)	.945 (.031)	.879 (.061)
$\pi /\tau _{nnny}$ (never A, former B)	.000 (.002)	.008 (.023)	.007 (.026)	.007 (.021)
$\pi /\tau _{nnyy}$ (never A, last year B)	.005 (.010)	.010 (.011)	.010 (.013)	.009 (.011)
$\pi /\tau _{ynnn}$ (former A, never B)	.000 (.001)	.001 (.022)	.002 (.017)	.001 (.021)
$\pi /\tau _{ynyn}$ (former A, former B)	.000 (.000)	.000 (.001)	.000 (.000)	.000 (.000)
$\pi /\tau _{ynyy}$ (former A, lastyear B)	.006 (.006)	.007 (.007)	.007 (.007)	.007 (.007)
$\pi /\tau _{yynn}$ (last year A, never B)	.006 (.009)	.009 (.011)	.009 (.012)	.008 (.010)
$\pi /\tau _{yyyn}$ (last year A, former B)	.000 (.000)	.004 (.007)	.004 (.007)	.004 (.006)
$\pi /\tau _{yyyy}$ (last year A, last year B)	.017 (.007)	.016 (.007)	.016 (.010)	.015 (.007)
$\theta /\tau _c\quad ~~$		.071 (.037)	.072 (.036)	.071 (.037)
$\theta _{yy\cdot \cdot ~\rightarrow ~yn\cdot \cdot }=\theta _{\cdot \cdot yy~\rightarrow ~\cdot \cdot yn}$			.023 (.353)	
goodness of fit	$G^2_{(7)}=9.46$	$G^2_{(6)}=4.31$	$G^2_{(5)}=4.30$	$G^2_{(1)}=4.31$
	$p=.222$	$p=.645$	$p=.507$	$p=.645$
AIC	3920.6	3917.4	3919.4	3917.4

## Discussion

By formulating CDM and SP-no models for the sub-samples and multiple question designs, we have shown the correspondence between the parameters of these models. We then introduced the ever/last year design as an alternative to these designs, and formulated models for this design and its extensions to multiple questions. By doing so, we provide practitioners with the tools to make a well-informed choice for a design. This choice should primarily be based on the purpose of the study, but it also involves considerations about the power to detect the prevalence of both the sensitive attribute(s) and evasive responses, and the trade-off between making untestable strong assumptions and the interpretability of the prevalence estimates as either point or interval estimates. Below we summarize the benefits and limitations that are relevant for the choice of a particular model and design.

Designs for a single sensitive attribute are the sub-samples and ever/last year design. The null models for these designs both have a degree of freedom, but they differ in the assumptions that have to make to estimate cheating/SP-no saying. The sub-samples design simply assumes that cheaters/SP-no sayers answer “no” to the sensitive question irrespective of the outcome of the randomizer, while the ever/last year design makes the additional strong assumption that cheaters/SP-no sayers do this to both questions. The benefit of the latter design is that it yields a more efficient estimate of the last year users than the sub-samples design. The models for these designs also differ in the assumptions they make with respect to the true responses of the cheaters/SP-no sayers. The CDM is assumption-free in the sense that it does not make any assumptions with respect to the true responses of the cheaters. As a consequence, the prevalence estimates of carriers and non-carriers are interval estimates, the width of which is determined by the prevalence estimate of the cheaters. The SP-no model, on the other hand, does make assumptions with respect to the true responses of the SP-no sayers, and therefore yields point estimates for the carriers and non-carriers that are corrected for SP-no saying.

In this paper we focused on CDM and SP-no models for the ever/last year design, and its extensions with a dichotomous question and another set of ever/last year questions. The main benefit of the extended designs is the increased power to detect cheating/SP-no saying. The power increase is exemplified by the data of Study I, where the models for the ever/last year questions on anabolics yielded an insignificant prevalence estimate of 4.6% for cheating/SP-no saying, while the model with the additional SARMs question yielded a significant estimate of 6.4%. A reviewer wondered whether this result could not alternatively be explained by a higher proportion of evasive responses to the SARMs question. We have included a simulation study in Appendix C to investigate this. The study shows that a higher proportion of evasive responses to the SARMs question does not bias the prevalence estimates of never, former and last year users of anabolics, and does not lead to a higher estimates of SP-no saying/cheating. It does however result in an underestimate for SARMs use. The reason for this is that the prevalence estimate for the SARMs question is free to take any value because its (univariate) model is saturated. In the model for the three questions the excess of evasive responses to the SARMs question will therefore be explained by an underestimate of the SARMs prevalence and will not substantially affect the estimate of SP-no saying/cheating.

An advantage of the SP-no model over the CDM is that it allows for the inclusion of additional response bias parameters. The SP(last year) model served as a somewhat speculative example of this. For the data of Study I, this model yielded contradictory results, with a highly significant estimate of the parameter $\theta _{yy\cdot ~\rightarrow ~yn\cdot }$, but also a higher AIC than the competing models. It is not clear whether these results indicate that the parameter $\theta _{yy\cdot ~\rightarrow ~yn\cdot }$ indeed detected some last year users that answered evasively to the last year question, or that the model simply overfitted the data. In the data of Study II, however, provided no evidence for the SP(last year) assumption. Future studies may provide a more definite answer to the question whether last year carriers are indeed inclined to answer “no” to the last year question when they already answer “yes” to the ever question.

A final word on the interpretation of the cheating/SP-no parameters. These parameters are sometimes erroneously interpreted as the proportion of misreported responses. This is incorrect, because a substantial fraction of the cheaters/SP-no sayers does not have to misreport because they have to answer “no” by design. As an illustration, consider a dichotomous question with $p_{n|n}=5/6$, so that five out of six non-carriers have to answer “no,” so that only one out of six of the SP-no sayers in this group has to answer “yes” and thus has to misreport. Analogously, five out of every SP-no sayers in the carriers group have to misreport. Given a prevalence of carriers of 0.1 and of SP-no sayers of 0.2, the total proportion of actually misreported responses in the entire sample is then computed as $(.2)(.9)(1/6)+(.2)(.1)(5/6)\approx 0.047$. In this example, less than one-quarter of the SP-no sayers had to misreport.

A closing remark concerns regression models for the ever/last year design. While there are numerous examples of regression models for the multiple questions design in the literature [e.g., Böckenholt and van der Heijden (2007)], such models have not yet been developed for the ever/last year design. Appendix D (https://osf.io/autr5/?view_only=2af4b338b9be45cd8657f926438c5f93) derives regression models for this design that allow for the explanation of both the prevalence of the sensitive attribute and the probability of cheating/SP-no saying in terms of covariates.

## Conclusion

This paper showed the correspondence between cheating and SP-no saying for the sub-samples and multiple questions design, and derived such models for a design with ever and last year questions about the same sensitive attribute. These models yield prevalence estimates of non-carriers, former carriers and last year carriers while at the same time allowing for the estimation of cheating/SP-no saying. We furthermore showed that by extending this design with questions about other sensitive attributes, alternative hypotheses about response bias can be tested through the inclusion of additional response bias parameters in the transition matrix. This allowed us to formulate a model that accounts for last year users who edit their response to the last year question when they already answered “yes” to the ever question, but we found no convincing evidence for this in our example data. For researchers who are more interested in detecting response biases than in the prevalence estimates of the sensitive attributes, we recommend to use randomization probabilities closer to 0.5, as our power study showed that this enhances the power to detect such biases. Obviously, this benefit comes at the expense of increased variances of the prevalence estimates of the sensitive attribute. The Achilles heel of the models for multiple questions is the strong assumption that a class of cheaters/SP-no sayers exists who consequently give an evasive “no” answer to all questions. While it seems difficult to test this assumption experimentally, future sensitivity analyses can provide insight in the effects on the parameter estimates when this assumption is not or only partially fulfilled.

## Data Availability

The dataset, appendix of all derivations, and the R codes necessary to reproduce the results are available on the OSF repository via: https://osf.io/autr5/?view_only=2af4b338b9be45cd8657f926438c5f93
